# Thymoquinone prevents valproic acid-induced hepatotoxicity via modulation of cytochrome P450, PPARs, and apoptotic pathways

**DOI:** 10.22038/ijbms.2025.85190.18407

**Published:** 2025

**Authors:** Sebile Azırak, Sedat Bilgiç, Deniz Taştemir Korkmaz, İlkay Armağan, Mehmet Özer Kaya

**Affiliations:** 1 University of Adiyaman, Vocational School of Health Services, Adiyaman, Turkey; 2 Department of Medical Biology, Faculty of Medicine, Adiyaman University, Adiyaman, Turkey; 3 University of Suleyman Demirel, Department of Histology and Embryology, Faculty of Medicine, Isparta, Turkey; 4 University of Adiyaman, Department of Pharmacology, Faculty of Medicine, Adiyaman, Turkey

**Keywords:** Apoptosis, Cytochromes, Liver, Oxidative stress, PPAR, Thymoquinone, Valproic acid

## Abstract

**Objective(s):**

Thymoquinone (TQ) is the main bioactive component of *Nigella sativa* L. and has anti-oxidant, anti-hepatotoxic, anti-cancer, anti-hypertensive, hypoglycemic, anti-inflammatory, and lipid-lowering properties. In this study, we investigated the protective properties of TQ on the cytochrome P450 enzyme system, peroxisome proliferator-activated receptors, and gene expressions involved in apoptosis, which are disrupted by valproic acid (VPA).

**Materials and Methods:**

The rats were put into control, VPA, and VPA+TQ groups. The weight of the body and liver were recorded. Liver tissue samples were evaluated for gene expressions (Bcl-2, p53, CYP2B1, CYP2B2, PPARα, and PPARγ), histopatology, and immunohistochemistry (CAS-3 and NOX-4). Additionally, serum was used to measure liver function parameters (ALT, AST, LDH, LDL, and HDL).

**Results:**

The VPA+TQ group had significantly lower expression of p53 (*P*<0.05), CYP2B1 (P<0.05), CYP2B2 (*P*<0.05), PPARα (*P*<0.05), and PPARγ (*P*<0.05) genes compared to the VPA groups, while Bcl-2 (*P*<0.05) gene expression increased. TQ decreased CAS-3 and NOX-4 levels. Also, TQ reduced ALT (*P*<0.05), AST (*P*<0.05), LDL (*P*<0.01), total bilirubin (*P*<0.05), and LDH (*P*<0.05) enzyme activity while increasing HDL (*P*<0.0001). TQ treatment improved fresh liver weight considerably (*P*<0.0001).

**Conclusion:**

TQ substantially protects liver tissue by modifying gene expression, lowering oxidative stress, and increasing liver function. It significantly counteracts VPA’s toxic effects, demonstrating its promise as a hepatoprotective agent in avoiding liver damage.

## Introduction

Valproic acid (VPA) is a widely used medicine with a wide range of therapeutic applications, including epilepsy treatment, bipolar disorder management, and migraine prevention in diverse seizure types in neurological and psychiatric illnesses (1). However, it is well recognized that VPA has numerous adverse effects in addition to its therapeutic benefits. VPA is harmful to the liver and other organs (2) and causes numerous side effects, including alopecia, pancreatitis, abdominal discomfort, thrombocytopenia, coagulation problems, hyperammonemic encephalopathy, and rhabdomyolysis (3). Although VPA treatment is frequently associated with liver impairment, the mechanisms underlying VPA-induced hepatotoxicity remain unknown. 

The cytochrome P450 (CYP) enzyme superfamily makes the liver the primary site of drug metabolism (4). Many subfamily enzymes within the CYP enzyme family can catalyze drugs and xenobiotic reactions (5). A wide range of medicines and substances can activate or inhibit CYP2B1 and CYP2B2, both of which have low substrate specificity (6). Induction and inhibition of CYP, crucial elements in drug biotransformation, are valuable indicators for assessing chemical compounds’ potential toxicity (7). 

Peroxisome proliferator–activated receptors (PPARs), ligand-activated transcription factors from the nuclear receptor superfamily, are involved in glucose and lipid metabolism. Therefore, they can be used as markers for both. The three PPAR isoforms, PPARα, PPARγ, and PPARβ/δ, have unique tissue distribution and functional roles (8). PPARs are found in the liver, heart, brain, skeletal muscle, kidney, and brown adipose tissue (9). PPARα reduces lipid levels and impacts fatty acid metabolism. PPARγ is involved in lipid biosynthesis, energy balance, and inflammatory regulation (10) and influences tumor growth, angiogenesis, cell differentiation, and apoptosis (11). PPARβ/δ modulates cholesterol and blood sugar levels and plays a role in fatty acid oxidation in skeletal and cardiac muscles (12). 

p53 is a transcription factor that controls the expression of several genes related to apoptosis, tumor suppression, cell cycle arrest, and aging (13). The B-cell lymphoma gene-2 (Bcl-2) gene family, which governs pro- and anti-apoptotic intracellular signals, is critical in controlling apoptosis (14). In response to numerous biological stresses, p53 causes DNA repair, aging, apoptosis, and cell cycle arrest (15), while Bcl-2 helps cells survive by preventing apoptosis (16).

Drug toxicity is a widespread issue nowadays, and in some circumstances, natural products are recommended as a supplement to traditional care. This study used thymoquinone (TQ), a natural substance, to lower VPA toxicity while maintaining its therapeutic efficacy. TQ is a bioactive component of *Nigella sativa* essential oil (17). TQ is derived from black cumin seed and has anti-oxidant, antihyperlipidemic, antimicrobial, anti-diabetic, anti-inflammatory, antihistamine, anticancer (18, 19), antiviral (20), gastroprotective, and hepatoprotective properties (21). 

In this study, we aimed to evaluate the potential role of TQ in mitigating the adverse effects of VPA on the CYPs, which are essential for liver metabolism, as well as PPARs, apoptosis-related genes, biochemical markers, and histopathological parameters.

## Materials and Methods

### Chemicals

VPA, TQ (purity > 98%), and the other chemicals utilized in the study were obtained from Sigma Aldrich Chemical Co. (St. Louis, MO). 

### Animals

Our experimental study was approved by the Fırat University Faculty of Medicine Ethics Committee (Protocol no. 2016/41). We received a total of 21, 3–4-month-old male Sprague-Dawley rats weighing 200–300 g from Fırat University Experimental Animal Research Center. The rats were kept at 21 **°**C with a 12 hr light:12 hr dark cycle and free access to food and water. All experimental protocols followed the National Institutes of Health Guidelines for the Care and Use of Laboratory Animals (NIH Publications No. 8023, revised 1978) and ARRIVE Guidelines.

### Experimental design

The 21 rats were randomly assigned to three groups of seven: the control group received saline solution, the VPA group received 500 mg/kg VPA, and the VPA + TQ group received 500 mg/kg VPA plus 50 mg/kg TQ. VPA and TQ were given orally once daily for 14 days. VPA (22) and TQ (23) dosages were determined using prior reports. On day 15, rats were sacrificed after receiving intramuscular injections of ketamine (30 mg/kg IM) and xylazine (5 mg/kg IM), and an intracardiac blood sample was collected using a syringe to evaluate liver enzyme activity. Serum samples were separated by centrifugation at 5,000 x g for 15 min and stored at -80 °C for biochemical analysis. The liver tissue was removed and weighed, then separated into two sections. One component was kept at −80 °C until gene expression analysis was performed. The remaining half was fixed in 10% neutral formalin for histological analysis. 

### Liver function assessment

Serum alanine aminotransferase (ALT) (U/l), aspartate aminotransferase (AST) (U/l), and lactate dehydrogenase (LDH) (U/l) enzyme activities, as well as high-density lipoprotein cholesterol (HDL) (mg/dl), low-density lipoprotein cholesterol (LDL) (mg/dl), and total bilirubin (TB) (mmol/l) levels were used to assess liver damage. The measurements were made using a spectrophotometric approach in the 340–380 nm wavelength range with an Abbott Labs Architect C16000 system (Abbott GmbH & Co., Wiesbaden, Germany) and commercial Abbott kits (24, 25). 

### Real-time PCR

Frozen tissue specimens were defrosted at +4 °C. Thirty milligrams of liver tissue from all rats were homogenized (Bioprep-24, Hangzhou Allsheng Instruments Co., Ltd., Hangzhou, China) in 500 µl of tissue lysis solution for one minute. Total RNA was extracted utilizing the ExiPrepTM Tissue Total RNA isolation kit (K-3325; Bioneer Inc., Oakland, CA, USA).

The RNA purity was assessed at 260–280 nm and 230–260 nm absorption wavelengths utilizing a NanoDrop spectrophotometer (Denovix DS-11; Denovix Inc., Wilmington, USA). RNA samples were subsequently transformed into cDNA with AccuPower^®^ RT PreMix (K-2041; Bioneer Inc., Oakland, CA, USA), following the manufacturer’s guidelines. The GAPDH gene was amplified to serve as an internal control. The mRNA expression levels of CYP2B1, CYP2B2, PPARα, PPARγ, Bcl-2, and p53 genes were assessed using the ExiCyclerTM96 Real-Time Quantitative PCR system (Bioneer Inc., Oakland, CA, USA) with the following protocol: thermal cycling at 95 ⁰C for five minutes, succeeded by 45 cycles at 95 °C for 15 sec, and subsequently at 60 °C for 25 sec (25). The primer sequences for CYP2B1, CYP2B2, PPARα, PPARγ, Bcl-2, and p53 (S-1001; Bioneer Inc.) are presented in Table 1. Gene expression levels were quantified utilizing the 2^-ΔΔCt^ methodology (26). 

### Histopathological examinations

Tissues preserved in 10% neutral formalin were rinsed with running water to eliminate surplus formalin, subsequently dehydrated with graded alcohols, clarified with xylene, and finally embedded in paraffin. Subsequently, they were sectioned at 3–4 μm using a rotary microtome (RM2125RTS; Leica, Nussloch, Germany) and adhered to slides with gelatin. Sections underwent deparaffinization, rehydration through graded alcohols, and subsequent staining with hematoxylin and eosin (H&E) for histological assessment (27). Histopathological alterations were evaluated for vacuolar degeneration in hepatocytes, mononuclear cell infiltration in the portal region and parenchyma, sinusoidal dilation, and vascular congestion. A modified semiquantitative scale was employed for assessment as follows: 0, no damage; 1, mild damage; 2, moderate damage; 3, severe damage (28). Samples were evaluated and imaged using an imaging-assisted binocular light microscope (Eclipse Ni-U; Nikon, Tokyo, Japan).

### Immunohistochemistry

Caspase-3 (CAS-3) and NADPH oxidase-4 (NOX-4) receptor activity was detected using immunostaining. Following deparaffinization and rehydration of the sections, they were subjected to treatment with 3% hydrogen peroxide (ScyTek Laboratories, Logan, UT, USA) and Super Block (ScyTek Laboratories) and subsequently incubated with primary antibodies for two hours. Primary antibodies sourced from Abcam (Cambridge, England) comprised anti-CAS-3 (06-735) at a dilution of 1:200 and anti-NOX-4 (ABC459) at a dilution of 1:50. Biotinylated goat anti-polyvalent (Abcam, Cambridge, England) served as the secondary antibody to interact with the primary antibodies. Horseradish peroxidase-conjugated streptavidin (ScyTek Laboratories) was utilized to bind with biotin, followed by the application of 3.3′-diaminobenzidine solution (ScyTek Laboratories) to colorize the receptor region. Subsequently, the sections were counterstained with Harris’ hematoxylin. The immunoreactivity of the histological preparations was semiquantified according to the degree of staining as follows: -, no staining; +, weak staining; ++, moderate staining; +++, intense staining. Ultimately, slides were seen employing imaging-assisted light microscopy (Eclipse Ni-U; Nikon) **(**28**)**.

### Statistical analysis

Statistical analysis was performed using Statistical Package 25.0 (SPSS, Chicago, IL, USA) and GraphPad Prism, version 9 software (GraphPad Software Inc., La Jolla, CA, USA). Data are presented as means ± SEM. One-way ANOVA with LSD was employed to evaluate data from group comparisons of parametric values for liver weight and genetic and biochemical markers. The Mann-Whitney U test was utilized to compare the histopathological and immunohistochemical results among groups (29). Differences were considered significant at *P*˂0.05. 

## Results

### Effects of VPA and TQ on liver weight

A notable reduction in the fresh liver weight to body weight ratio was seen in the VPA + TQ group relative to the VPA group (*P*˂0.0001) (Table 2).

### Effects of VPA and TQ on liver enzyme activities and biochemical marker levels

**Figure 1:**
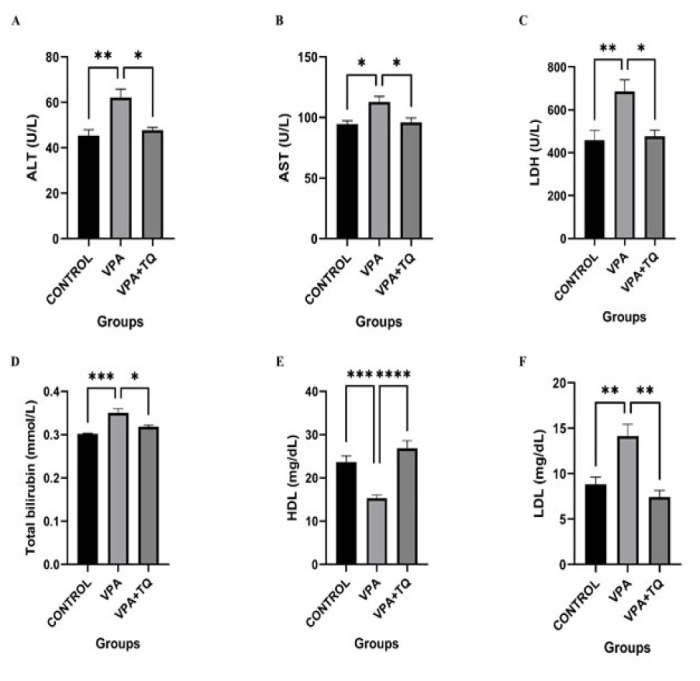
Effects of VPA and TQ on liver function markers in rats

The activity of ALT, AST, and LDH enzymes, along with the serum levels of LDL, HDL, and TB are presented in Figure 1. ALT (*P*˂0.01), AST (*P*˂0.05), LDH (*P*˂0.01), LDL (*P*˂0.01), and TB (*P*˂0.001) levels were considerably elevated in the VPA group relative to the control group, but HDL (*P*˂0.001) levels were dramatically diminished. Conversely, enzyme activities of ALT (*P*˂0.05), AST (*P*˂0.05), and LDH (*P*˂0.05), as well as LDL (*P*˂0.01) and TB (*P*˂0.05), were significantly reduced in the VPA + TQ group compared to the VPA group, whereas HDL (*P*˂0.0001) levels were dramatically elevated (Figure 1).

### Effects of VPA and TQ on the expression of CYP2B1 and CYP2B2 genes

**Figure 2:**
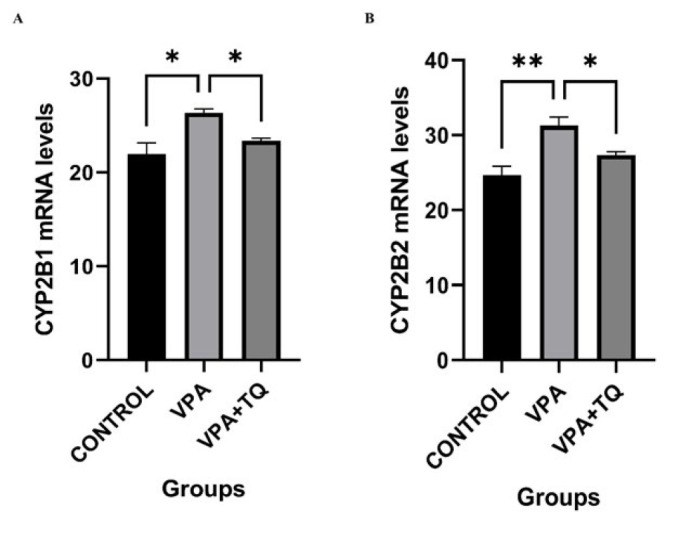
Effects of VPA and TQ on CYP2B1 and CYP2B2 mRNA expressions in rat liver

Figure 2 illustrates the impact of TQ treatment on the mRNA expression levels of the CYP2B1 and CYP2B2 genes across all groups subsequent to VPA administration. The expressions of CYP2B1 (*P*˂0.05) and CYP2B2 (*P*˂0.01) genes were considerably elevated in the VPA group relative to the control group. The VPA + TQ group had a markedly reduced expression of CYP2B1 (*P*˂0.05) and CYP2B2 (*P*˂0.05) genes in comparison to the VPA group (Figure 2). 

### Effects of VPA and TQ on the expression of PPARα and PPARγ genes

**Figure 3:**
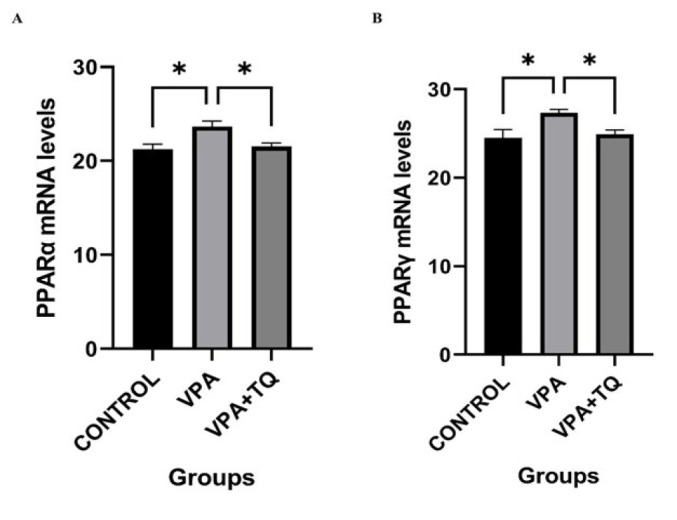
Effects of VPA and TQ on PPARα and PPARγ mRNA expressions in rat liver

Figure 3 illustrates the impact of TQ treatment on the mRNA expression levels of PPARα and PPARγ genes across all groups subsequent to VPA administration. Gene expressions of PPARα (*P*˂0.05) and PPARγ (*P*˂0.05) were considerably elevated in the VPA group relative to the control group. The VPA + TQ group exhibited a notable enhancement in the expression of PPARα (*P*˂0.05) and PPARγ (*P*˂0.05) genes relative to the VPA group.

### Effects of VPA and TQ on the expression of Bcl-2 and p53 genes

**Figure 4:**
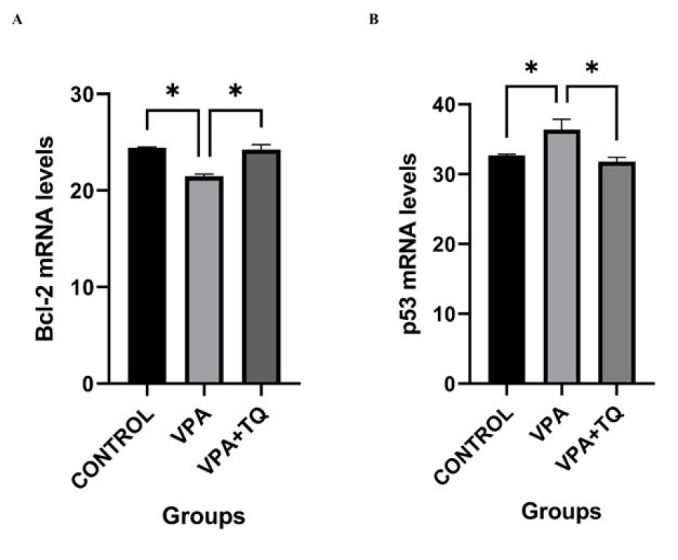
Effects of VPA and TQ on Bcl-2 and p53 mRNA expression in rat liver

Figure 4 illustrates the impact of TQ treatment on the mRNA expression levels of the Bcl-2 and p53 genes across all groups subsequent to VPA administration. In the VPA group, Bcl-2 gene expression dramatically decreased (*P*<0.05) relative to the control group, whereas p53 gene expression significantly increased (*P*<0.05) compared to the control group. The VPA + TQ group demonstrated a significantly elevated Bcl-2 gene expression (*P*<0.05) compared to the VPA group. The VPA + TQ group exhibited a significant reduction in p53 gene expression (*P*<0.05) relative to the VPA group (Figure 4). 

### Histopathological findings

**Figure 5:**
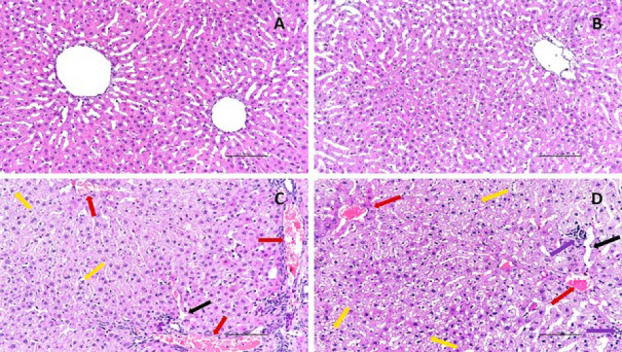
Rat liver tissue section

The control group exhibited nearly normal liver histology (Figure 5A). In the VPA group, we observed many instances of vacuolar degeneration in hepatocytes, mononuclear cell infiltration in the parenchyma and portal region, sinusoidal dilation, and moderate vascular congestion (Figure 5C, D). Histopathological alterations, including degeneration, infiltration, and vascular dilation, were less prevalent in the VPA + TQ group (Figure 5B). 

### Immunohistochemistry

**Figure 6:**
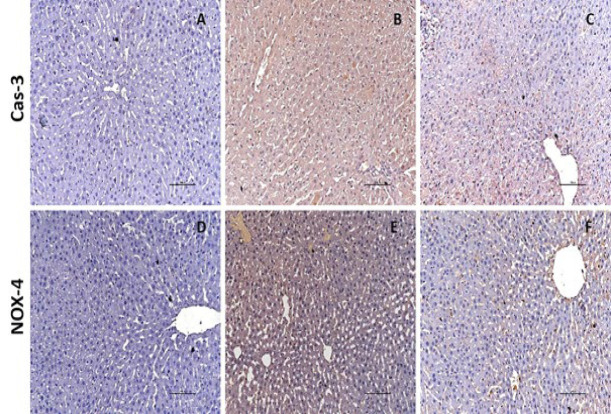
Immunoreactivity of CAS-3 and NOX-4

Figure 6 encapsulates the immunoreactivity results for CAS-3 and NOX-4. The control group displayed no antibodies for CAS-3 and NOX-4 in liver tissue (Figure 6A, 6D). Pronounced CAS-3 positivity was noted in the VPA group, whereas NOX-4 immunoreactivity was moderate (Figure 6B, 6E). In the VPA + TQ group, immunoreactivity for CAS-3 and NOX-4 was diminished (Figure 6C, 6F). 

## Discussion

VPA, an anti-epileptic drug, is used clinically to treat diseases such as epilepsy and psychiatric disorders (30). In studies conducted on this subject, including long-term VPA use, it was found that liver functions were impaired. Moreover, damages resulting from VPA use include hepatic encephalopathy, chronic liver failure, hyperammonemia, and Reye-like syndrome (31). It is known that the primary source of hepatotoxicity caused by VPA in the liver is oxidative stress. The factors leading to this situation include decreased anti-oxidants, depletion of mitochondrial DNA, hypermethylation, oxidative phosphorylation disorder, and decreased ATP synthesis (32). In patients receiving VPA treatment, reducing side effects and providing alternatives that support the treatment can increase the chance of success. In addition, supportive herbal products can help these patients enhance their quality of life, mitigate adverse effects, and strengthen their immune systems. In this context, the use of herbal compounds is increasing along with VPA treatment (33, 34). It is known that the natural compound TQ is among the most suitable alternatives in this regard.

A number of adverse effects occur in epileptic and psychiatric patients treated with VPA, including significant weight gain and elevated body mass index (35). It has been determined that these patients who gain weight as a result of this treatment have insulin resistance and leptin resistance problems. Moreover, some studies have reported that these patients also have hyperinsulinemia and hyperleptinemia (36). It has been shown that liver size can change when exposed to xenobiotics or injury, and PPARα, in particular, can induce liver growth (37). Our findings are consistent with these reports, supporting the observed effects of VPA and TQ on weight gain and liver size. 

As it is widely known, serum levels of ALT, AST, HDL, LDH, LDL, and TB are important indicators of liver damage. In our study, the VPA group also showed significantly elevated ALT, AST, LDH, LDL, and TB parameters, consistent with an earlier report (38). According to the data on this subject in the literature, there may be many reasons for the high levels of enzymes and TB detected in serum. These reasons include enzyme leakage from the cytosol into the serum as a result of cell membrane damage and necrosis in hepatocytes (39). In addition, an increase in ALT and AST enzyme levels in the serum has been interpreted as indicative of potential hepatotoxic effects of VPA. The dual administration of TQ and VPA resulted in substantial reductions in the levels of these enzymes. This phenomenon can be ascribed to TQ’s ability to maintain cell membranes, inhibiting enzyme passage into the serum. Additionally, we observed a significant increase in serum LDL levels and a drop in HDL levels in the VPA group. TQ may offer protection against VPA-induced liver injury due to its reactive oxygen species (ROS) scavenging and lipid-lowering properties (40). The findings we acquired demonstrate that TQ therapy may be beneficial in mitigating the hepatotoxicity that arises.

VPA therapy elevated the expression of CYP2B1 and CYP2B2 genes. The liver is essential for metabolic activities, rendering it susceptible to damage. Researchers have demonstrated that this state is frequently induced by the activation of several cytochromes, notably CYP2B1 and CYP2B2 (41). Our findings align with research indicating that CYP2B1/B2 upregulation leads to oxidative stress and apoptosis due to insufficient intracellular anti-oxidant defenses, resulting in liver injury (42, 43). The activity of anti-oxidant enzymes is essential for cellular detoxification from xenobiotics, deleterious substances, and oxidative stress (44). Consequently, our research used TQ, an anti-oxidant, in conjunction with VPA. The VPA + TQ group demonstrated markedly reduced expression of CYP2B1 and CYP2B2 genes compared to the VPA group.

PPARs are transcription factors that regulate various biological processes, including general energy homeostasis, lipid and glucose metabolism, and inflammation. They are regarded as novel therapeutic targets for understanding adipogenesis and metabolic disorder treatments (45). Our study results indicate a considerable rise in the expression of PPARα and PPARγ genes. Our findings are consistent with studies showing that VPA disrupts lipid metabolism in the liver by interacting with PPARs, causes abnormal lipid retention in hepatocytes, and eventually leads to liver injury due to lipid accumulation (46, 47). A study demonstrated that oxidative stress, apoptosis, and inflammation caused by lipid metabolism disturbance exacerbate liver damage by promoting lipid peroxidation (48). Our findings are consistent with the study results showing that TQ interacts with amino acids in the ligand-binding pocket of PPARs and mitigates hepatotoxicity via regulating PPARα and PPARγ (49). Our study indicates that, despite the hepatotoxic effects of VPA, TQ mitigates lipid peroxidation by alleviating oxidative stress due to its anti-oxidant qualities and diminishes liver damage through its modulatory effects on PPARs that govern lipid metabolism.

We assessed the impact of VPA and TQ therapy on p53, Bcl-2, CAS-3, and NOX-4 levels. VPA therapy reduced Bcl-2 gene expression while elevating p53, CAS-3, and NOX-4 expression levels. The findings of studies indicating that elevated p53 expression reduced Bcl-2 expression and induced apoptotic cell death due to hypoxia and DNA damage align with our results (50, 51). Our results align with the data indicating that oxidative stress induced by VPA therapy elevates apoptosis and reduces Bcl-2 gene expression levels (52). In our study, CAS-3 immunoreactivity, an indicator of apoptosis in hepatic tissues, was missing in the control group, but intense immunostaining was noted in the VPA group. The elevation of CAS-3 activity in normal hepatic tissues and the initiation of apoptosis can be ascribed to VPA treatment. NOX-4 is involved in the regulation of ROS generation and DNA damage (53). The substantial elevation of NOX-4 levels in the VPA group suggests that VPA induces oxidative stress in the liver. This results in the suppression of cell growth and irreversible apoptosis, accompanied by elevated p53 expression (54). Our findings suggest that VPA induced tissue damage by instigating oxidative harm and initiating inflammatory and apoptotic processes (55). Consistent with our findings, a prior study noted the direct relocation of p53 to mitochondria, cytochrome c release, reductions in Bcl-2 levels, and elevations in CAS-3 activation subsequent to DNA damage (56). Our findings align with literature research indicating heightened oxidative stress due to elevated NOX-4 expression. Apoptosis induction is evidenced by elevated cytochrome c release resulting from diminished Bcl-2 expression (57). In conclusion, our data indicate that oxidative stress caused by VPA might trigger a cascade of detrimental events, resulting in cell death.

Our work demonstrates that the reduction in p53 gene expression, the considerable decrease in CAS-3 and NOX-4 levels, and the notable enhancement in Bcl-2 gene expression in the VPA + TQ group validate the efficacy of TQ. The weak CAS-3 immunostaining observed in the TQ-treated group suggests that TQ treatment inhibits apoptosis by averting the elevation of CAS-3 levels in normal hepatic tissue impacted by VPA. Moreover, TQ’s suppression of NOX-4 levels may be ascribed to the attenuation of VPA-induced oxidative stress and apoptosis. Based on the data collected, it can be concluded that TQ administration in our study diminishes apoptosis and oxidative stress, consistent with other research (58).

Our study’s main VPA-induced histopathological changes were vacuolar degeneration in hepatocytes, mononuclear cell infiltration in the parenchyma and portal area, sinusoidal dilation, and vascular occlusion. The data we obtained are also compatible with the literature on this subject (59, 60). In our study, the significant improvements in histopathological changes in the VPA + TQ group confirm that TQ application is effective. In addition, studies on this subject confirm that TQ provides significant improvement in hepatotoxicity (61). In light of all these findings, it can be stated that TQ has a hepatoprotective effect against VPA-induced liver injury.

**Table 1:** Nucleotid sequences of primers

**Genes**	**Sequence (5’–3’)**
*CYP2B1 *	Forward 5’-AACCCTTGATGACCGCAGTAAA-3’ Reverse 5’-TGTGGTACTCCAATAGGGACAAGATC-3’
*CYP2B2 *	Forward 5′-GGACACTGAAAAAGAGTGAAGCTTT-3’ Reverse 5′-AATGCCTTCGCCAAGACAAA-3’
*PPARα*	Forward 5′-CCATACAGGAGAGCAGGGATT-3′ Reverse 5′-CCACCATTTCAGTAGCAGGA-3′
*PPARγ*	Forward 5’- GACCACTCCCATTCCTTT-3’ Reverse 5’- GCTCTACTTTGATCGCACT-3’
*Bcl-2*	Forward 5′-AGGATAACGGAGGCTGGGATG-3′ Reverse 5′-TATTTGTTTGGGGCAGGTCT-3′
*P53*	Forward 5′-ATTTCACCCTTAAGATCCGTGGG-3′ Reverse 5′-AGACTGGCCCTTCTTGGTCT-3′
***GAPDH***	**Forward 5'- CAACTCCCTCAAGATTGTCAGCAA-3'** **Reverse, 5'-GGCATGGACTGTGGTCATGA-3'**

**Table 2:** Comparison of fresh liver weight and fresh liver weight/body weight ratio between groups

	Control	VPA group	VPA + TQ group
Fresh liver weight (g)	11.66 ± 0.35^c^	11.29 ± 0.46^c^	9.18 ± 0.88^a,b^
Fresh liver weight/body weight ratio	0.041 ± 0.001	0.042 ± 0.001^c^	0.039 ± 0.002^b^

Data are means ± SEM for seven rats. ^a^Significantly different from control.^ b^Significantly different from VPA group. ^c^Significantly different from VPA + TQ group. *P*<0.05.

VPA: valproic acid; TQ: thymoquinone

A: ALT, B: AST, C: LDH, D: Total bilirubin, E: HDL, F: LDL. VPA: valproic acid; TQ: thymoquinone; ALT: alanine transaminase; AST: aspartate transaminase; HDL: high-density lipoprotein; LDH: lactate dehydrogenase; LDL: low-density lipoprotein; TB: total bilirubin. ****P˂0.0001, ***P˂0.001, **P˂0.01, *P˂0.05.

Data are means ± SEM for CYP2B1 and CYP2B2 mRNA expressions compared to the expression of the GAPDH housekeeping gene. VPA: valproic acid; TQ: thymoquinone; CYP2B1: cytochrome P450 2B1; CYP2B2: cytochrome P450 2B2. **P˂0.01, *P˂0.05.

Data are means ± SEM for PPARα and PPARγ mRNA expressions compared to the expression of the GAPDH housekeeping gene. VPA: valproic acid; TQ: thymoquinone; PPARα: peroxisome proliferator-activated receptor α; PPARγ: peroxisome proliferator-activated receptor γ. *P˂0.05.

Data are means ± SEM for Bcl-2 and p53 mRNA expressions compared to expression of the GAPDH housekeeping gene. VPA: valproic acid; TQ: thymoquinone; Bcl-2: B cell lymphoma-2; p53: tumor suppressor. *P˂0.05.

A: Histological appareance of the liver tissue in control group, B: Mild histopathological changes with restoration of normal histological structures of the liver tissue in VPA + TQ group, C-D: Vacuolar degeneration in hepatocyte (yellow arrow), mononuclear cell infiltration in parenchyma and portal area (purple arrow), sinusoidal dilation (black arrow), vascular congestion (red arrow) in VPA group. VPA: valproic acid; TQ: thymoquinone. H &E staining. Scale bar =100 µm. × 200.

CAS-3 immunostaining; A: No staining in control group, B: Intense immunostaining in VPA group, C: Weak staining in TQ treated group. NOX-4 immunostaining; D: No staining in control group, E: Moderate staining in VPA group, F: Weak staining in TQ treated group. VPA: valproic acid; TQ: thymoquinone; CAS-3: Caspase 3; NOX-4: NADPH oxidase-4. Scale bar = 100 µm. x 200.

## Conclusion

TQ is interpretable as hepatoprotective against oxidative stress due to its many features, particularly its anti-oxidant capabilities. Our research encompasses genetic, biochemical, and histological evidence substantiating this TQ concept. Consequently, it has been established that TQ may offer protection against VPA-induced hepatotoxicity.

## Data Availability

The data supporting this study’s findings are available upon request from the corresponding author.

## Declaration

We have not used any AI tools or technologies to prepare this manuscript.
